# Vaccination of Gilthead Seabream After Continuous Xenoestrogen Oral Exposure Enhances the Gut Endobolome and Immune Status *via* GPER1

**DOI:** 10.3389/fimmu.2021.742827

**Published:** 2021-10-14

**Authors:** Pablo Castejón, Isabel Cabas, Victoria Gómez, Elena Chaves-Pozo, Isabel Cerezo-Ortega, Miguel Ángel Moriñigo, Eduardo Martínez-Manzanares, Jorge Galindo-Villegas, Alfonsa García-Ayala

**Affiliations:** ^1^ Department of Cell Biology and Histology, Regional Campus of International Excellence “Campus Mare Nostrum”, University of Murcia, Instituto Murciano de Investigacion Biosanitaria (IMIB), Centro de Investigacion Biomedica en Red Enfermedades Raras (CIBERER), Murcia, Spain; ^2^ Aquaculture Department, Oceanographic Center of Murcia, Spanish Institute of Oceanography (IEO-CSIC), Murcia, Spain; ^3^ Department of Microbiology, Faculty of Sciences, University of Malaga, Málaga, Spain; ^4^ Faculty of Biosciences and Aquaculture, Nord University, Bodø, Norway

**Keywords:** 16S rRNA, endocrine disruptors (EDCs), endobolome, estrogens, fish, G protein-coupled receptor 1, vaccination, vitellogenin (VTG)

## Abstract

In fish culture settings, the exogenous input of steroids is a matter of concern. Recently, we unveiled that in the gilthead seabream (*Sparus aurata*), the G protein-coupled estrogen receptor agonist G-1 (G1) and the endocrine disruptor 17α-ethinylestradiol (EE_2_) are potent modulators in polyreactive antibody production. However, the integral role of the microbiota upon immunity and antibody processing in response to the effect of EE_2_ remains largely unexplored. Here, juvenile seabreams continuously exposed for 84 days to oral G1 or EE_2_ mixed in the fish food were intraperitoneally (i.p.) immune primed on day 42 with the model antigen keyhole limpet hemocyanin (KLH). A critical panel of systemic and mucosal immune markers, serum VTG, and humoral, enzymatic, and bacteriolytic activities were recorded and correlated with gut bacterial metagenomic analysis 1 day post-priming (dpp). Besides, at 15 dpp, animals received a boost to investigate the possible generation of specific anti-KLH antibodies at the systemic and mucosal interphases by the end of the trial. On day 43, EE_2_ but not G1 induced a significant shift in the serum VTG level of naive fish. Simultaneously, significant changes in some immune enzymatic activities in the serum and gut mucus of the EE_2_-treated group were recorded. In comparison, the vaccine priming immunization resulted in an attenuated profile of most enzymatic activities in the same group. The gut genes qPCR analysis exhibited a related pattern, only emphasized by a significant shift in the EE_2_ group’s *il1b* expression. The gut bacterial microbiome status underwent 16S rRNA dynamic changes in alpha diversity indices, only with the exposure to oral G1, supporting functional alterations on cellular processes, signaling, and lipid metabolism in the microbiota. By the same token, the immunization elevated the relative abundance of *Fusobacteria* only in the control group, while this phylum was depleted in both the treated groups. Remarkably, the immunization also promoted changes in the bacterial class Betaproteobacteria and the estrogen-associated genus *Novosphingobium*. Furthermore, systemic and mucosal KLH-specific immunoglobulin (Ig)M and IgT levels in the fully vaccinated fish showed only slight changes 84 days post-estrogenic oral administration. In summary, our results highlight the intrinsic relationship among estrogens, their associated receptors, and immunization in the ubiquitous fish immune regulation and the subtle but significant crosstalk with the gut endobolome.

## Introduction

The endocrine system of multicellular organisms comprises secretory glands that release hormones that can regulate signal transduction pathways with exquisite specificity upon paracrine engagement with a specific cognate receptor in the target cells. These interactions lead to the modulation of a vast set of functions, including but not limited to energy balance metabolism, body weight regulation, growth development, reproduction, and immunity (1, 2). Alterations of the natural endocrine hormonal homeostatic balance eventually occur through the exposure to environmental single or mixed synthetic chemical pollutants exhibiting hormone-mimetic activities, collectively regarded as endocrine disruptor compounds (EDCs) (3, 4). However, endocrine disruption is not considered a toxicological endpoint *per se* but a functional change that may lead to adverse effects in the organism.

Estrogen plays an essential role in regulating immune responses through innate immune signaling modulation and the impairment of B-cell functions (5, 6). The natural estrogenic steroid 17β-estradiol (E_2_) and the xenoestrogen, the synthetic oral contraceptive 17α-ethinylestradiol (EE_2_), are two EDCs generally present in wastewaters that adversely affect aquatic organism and human health (7). The estrogenic ligands or compounds exert their canonical biological processes *via* the intracellular nuclear estrogen receptors (ERs) acting as the ligand-activated transcription factor, which binds to the estrogen-responsive element located within the promoter region of target genes (8). However, they can also rapidly activate transduction pathways *via* non-genomic mechanisms mediated by a membrane-anchored receptor called the G protein-coupled estrogen receptor 1 (GPER1) (9). Accumulating evidence indicates that both ERs and GPER1 mediates feedback loops or crosstalk among several complex signaling axes like the insulin-like growth factor-1 receptor/phosphatidylinositol 3-kinase–threonine protein kinase B–mammalian target of rapamycin (IGF-1R/PI_3_K–Akt–mTOR) (10), epidermal growth factor receptor/extracellular signal-regulated kinase 1/2 (EGFR/ERK1/2) (11), cyclic adenosine monophosphate/protein kinase A (cAMP/PKA) (12), reactive oxygen species/calcium-apoptosis signal-regulating kinase 1-c-Jun N-terminal kinase $\left(\text{ROS}/\text{C}{\text{a}}_{2}^{+}–\text{ASK}1–\text{JNK}\right)$ (13), interferon (IFN), or the apoptotic pathway (14). Therefore, to examine the influences of particular EDCs and distinguish GPER1-mediated estrogen action from the classic ER activation, a nonsteroidal, high-affinity, and selective GPER1 agonist G-1 (G1) was developed (15, 16).

As most vertebrates, teleost fish are subjected to developmental immunologic programming triggered by the action of diverse microbiota inhabiting the surrounding environment (17–19). Particularly, growing evidence sheds light on the profound impact that some bacteria have in the gut and *vice versa* on the modulation of many long-lasting biological processes and functions, such as metabolism, inflammation, immune, and stress responses (20–25). Besides, it has been acknowledged that endogenous steroid hormones and EDCs interact with the gut microbiota through different pathways (26). By combining a myriad of previous findings, two novel concepts were recently coined. Estrobolome refers to the enteric bacterial species possessing β-glucuronidase and β-glucosidase enzymes involved in the deconjugation of endogenous estrogen at the gut level (27, 28). Furthermore, the expanded term endobolome includes the gut microbiota that can metabolize also the synthetic EE_2_ (29).

Our former studies on the role of sex steroids in teleost fish immunity revealed that gilthead seabream (*Sparus aurata*) leukocytes express nuclear ERs and GPER1. GPER1 modulates leukocyte functions through a cAMP/protein kinase A/CREB signaling pathway (30, 31). We also provided evidence that EDCs altered the immune response by promoting long-lasting effects even when their disruptive estrogenic effects were not present (32, 33). Moreover, EE_2_ bath-exposed specimens have an altered capacity to respond to an immune challenge, even though the compound does not behave as an immunosuppressor. Simultaneously, the oral intake of the same compound may stimulate antibody response and promote the extended production of natural neutralizing antibodies mediated through GPER1 (34). In addition, efforts on understanding the effect of keyhole limpet hemocyanin (KLH) as an immunogenic protein antigen model in gilthead seabream under diverse scenarios have been extensively conducted (32, 35, 36). Nevertheless, the impact of oral EE_2_ exposure following a vaccination scheme targeting the adaptive immunity and relying on the generation of better and stronger responses after a primary immunization on the gilthead seabream-associated microbiome has never been explored before.

Therefore, this study aimed to investigate, *in vivo*, the effects resulting from supplementing the diet with exogenous EE_2_ and the agonist G1 in the gilthead seabream. Specifically, changes in the innate immune response were determined by quantifying the activity of humoral lytic and oxidative stress mediators. This study measured essential mucosal inflammatory marker genes, observed how goblet cells contribute to immunity at the gut mucosal surface, explored the microbiome landscape on the immune repertoire following priming immunization with KLH, and assessed the specific long-term systemic and mucosal antibody production under the proposed scheme. In summary, the results highlighted the intrinsic relationship between xenoestrogens and their associated receptors in the ubiquitous fish immune regulation, and the subtle but significant crosstalk with the fish gut endobolome is described here for the first time.

## Materials and Methods

### Fish and Trial Setup

Ninety-six healthy juvenile European gilthead seabreams (*S. aurata*) were obtained from the Oceanographic Center of Murcia (Mazarrón, Spain). The fish were randomly divided into three treatment groups (n = 32 fish/treatment), and subsequently, each group was redistributed into a replicate tank. The animals were stocked in fiber-reinforced plastic tanks of 200 L at the same facility where they were obtained and provided with running seawater (dissolved oxygen 6 ppm) with continuous water renewal (flow rate 20% aquarium vol/h) and under natural temperature (21.0°C ± 3.0°C) and photoperiod. Before the start of the trial, the fish underwent a period of acclimatization of 2 weeks. The fish were fed thrice daily with a commercial pellet diet (44% protein and 22% lipids: Skretting) until apparent satiation. The environmental parameters, mortality, and behavioral changes were recorded daily.

### Experimental Design

Following the acclimatization period, the fish were maintained by feeding on the basal (control) or supplemented diets (G1 or EE_2_) for 3 months continuously. Briefly, 98% pure EE_2_ (Sigma-Aldrich) or G1 (Tocris) was supplemented on the commercial feed at a single dose of 5 μg/g food using the ethanol evaporation method (0.3 L EtOH/kg of food), as described elsewhere (38). To evaluate the effect of G1 and EE_2_ under an immunization program, specimens were intraperitoneally (i.p.) primed and 15 days later boosted with a model vaccine containing KLH (100 μg/fish; Sigma-Aldrich) and Imject Alum adjuvant (4 mg/fish; Thermo Scientific) (vaccinated/immunized fish) or phosphate-buffered saline (PBS) (unvaccinated fish) at 42 and 56 days of treatment. Please note that we have previously reported that Imject alum enhances the specific antibody levels to the model antigen KLH in gilthead seabream (35, 39). Strikingly, however, Imject alone fails to promote mature *il1b* release or enhance specific antibody levels compared to the complete KLH model vaccine. Therefore, the KLH- or alum-only groups were omitted to optimize economic resources on sequencing in the present experimental setting.

### Sample Collection

**Figure 1:**
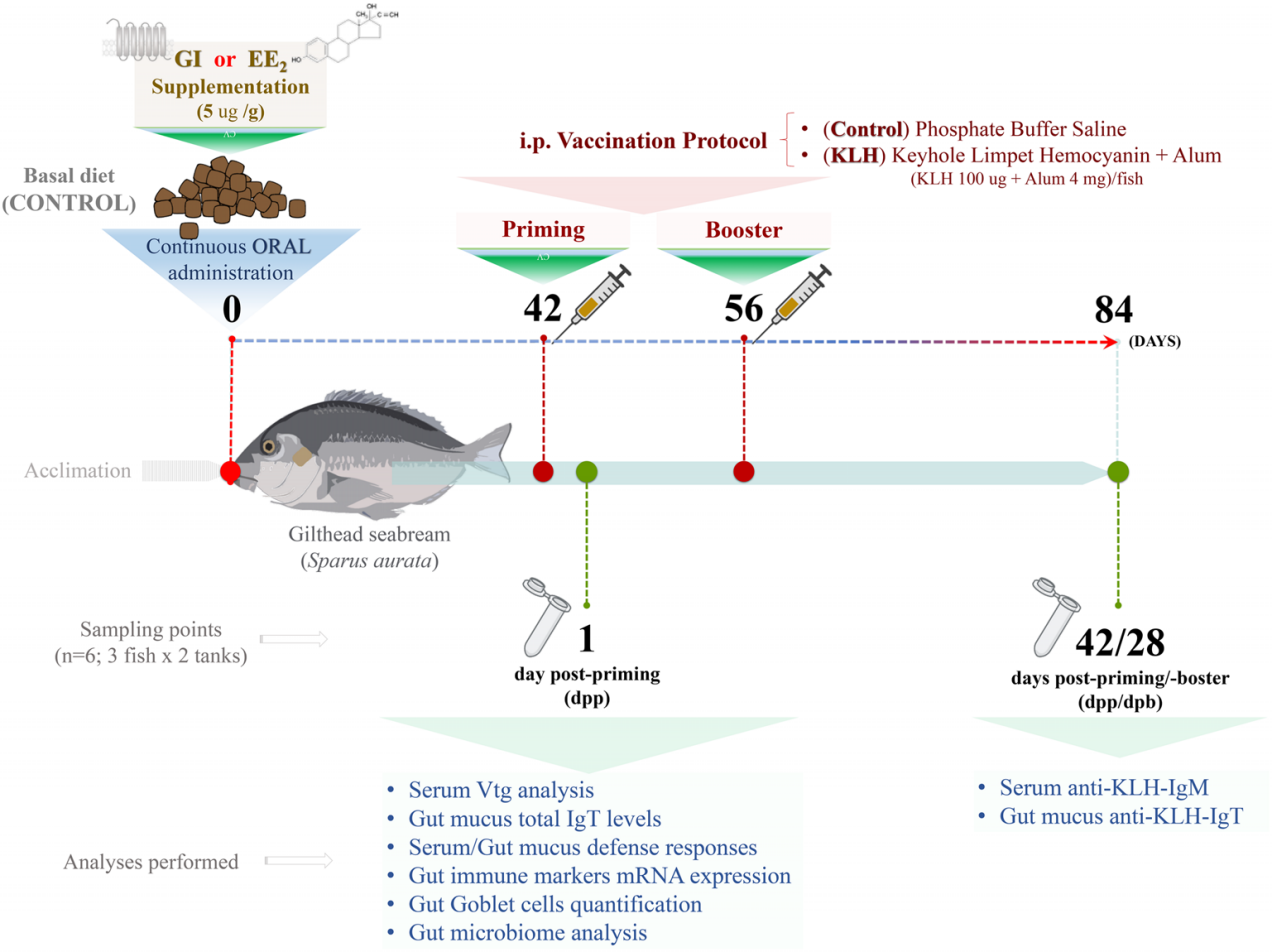
Experimental setup. From day 0, control or supplemented diet with G1 or EE2, orally administered daily within 84 days (12 weeks) to gilthead seabream. After 6 weeks, on day 42, half of the animals from each group were immune primed and boosted 15 days later (8 weeks) by intraperitoneal injections (i.p.) with repeated doses of the model vaccine containing KLH as antigen. The analyses in the list were conducted at 1 and 42 days after receiving the priming immunization (6 and 12 weeks, respectively). N=6; three fish from each duplicated tank per treatment.

As described in **Figure 1**, complete sets of samples were obtained 1 day post-priming (dpp) and 28 days post-booster coincident with the end of the trial on day 84. Prior to the sample collection, the fish were fasted for 24 h. Briefly, six specimens per treatment (three fish from each duplicated tank) and sampling point were sacrificed in less than 1 min through anesthetic (clove oil) overdose, and blood was collected from the caudal vein with 25- G needles attached to 2-ml syringe (40). The blood samples were allowed to clot for 1 h at room temperature, centrifuged (10,000g for 10 min), and the sera were collected and stored at -80°C until analysis. Meanwhile, the total gut mucus samples were collected using a sterile cell scraper and centrifuged at 3,000 for 1 min at 4°C to remove cells and debris. To separate bacteria from mucus, the cell-free supernatant was thereafter centrifuged at 10,000g for 10 min. The resulting supernatants were filtered through a 0.45-μm filter in order to detect the antibodies unbound to bacteria as explained elsewhere (41). In addition, the head-kidney and gut tissue samples free of fecal contents were also collected following standard aseptic procedures. Half of each sample obtained was immediately stabilized and protected from degradation by immersing it in 1.5-ml Eppendorf tubes prefilled with 1 ml of RNAlater solution (Thermo Fisher), while the other half was subjected to histological procedures as detailed further.

### Determination of Serum Vitellogenin Levels

The serum vitellogenin (VTG) levels were quantified by an enzyme-linked immunosorbent assay (ELISA) using a commercial kit (Cayman Chemical), following the manufacturer’s instructions, as previously described (34). In brief, an aliquot of 1:500 diluted serum from both the control and EE_2_- or G1-treated fish (n = 6 fish/treatment) was added to a flat-bottomed 96-well plate, followed by a commercial polyclonal antibody against gilthead seabream VTG and an anti-rabbit immunoglobulin (Ig)G peroxidase (Sigma-Aldrich), both diluted at 1:1,000 and incubated at 25°C for 1 h. Finally, the chromogen tetramethylbenzidine (TMB) was added, and the absorbance was read at 450 nm using a SPECTROstart nano (BGM; LabTechnologies).

### Detection of Serum and Gut Mucus Humoral Immune Parameters

Peroxidase activity was determined according to a previously described and adapted protocol for fish (42). One unit was defined as the amount of activity producing an absorbance change of 1, and the data were expressed as a fold increase relative to the mean of untreated and unvaccinated fish.

Antiprotease activity was determined by serum or gut mucus ability to inhibit the hydrolysis of azocasein by proteinase K activity (2 mg/ml) as previously suggested (43). The percentage of inhibition of proteinase K activity for each sample was calculated as [100- (% of protease activity)]. Results were expressed as a fold increase relative to the mean of untreated and unvaccinated fish.

Lysozyme activity was measured using a previously described protocol (35). Briefly, the enzyme activity was quantified according to a turbidimetric method that uses the lysis of *Micrococcus lysodeiticus* ATCC No. 4698 (Sigma-Aldrich) with hen egg-white lysozyme as the standard. One unit of lysozyme activity was defined as a reduction in absorbance at 450 nm of 0.001/min. Results were expressed as the fold increase relative to the mean of untreated and unvaccinated fish.

Bactericidal activity was assessed by evaluating their effects on the bacterial growth of *Vibrio harveyi* curves as described elsewhere (44). The pathogenic marine bacteria *V. harveyi* (strain Lg 16/100) was grown and cultured as previously described (45). Results were corrected with the absorbance measured in each sample at the initial time point and expressed as a fold increase relative to the mean of untreated and unvaccinated fish.

### Determination of IgM-Specific Antibody Levels

The hemocyanin-specific IgM level was determined by an ELISA kit (Aquatic Diagnostic, Ltd.) as previously described (35). Briefly, PBS as blank or fish sera from naive, hemocyanin-immunized, or pre-immune animals as control diluted 1:100 were added to the hemocyanin precoated 96-well ELISA plates, followed by the monoclonal antibody anti-seabream IgM at 1:1,000. Then, an anti-mouse IgG peroxidase antibody produced in goat (Sigma-Aldrich) diluted 1:1,000 was included as a reporter. Finally, the chromogen TMB (Sigma-Aldrich) was added, and the plates were incubated at 22°C. The reaction was stopped with the addition of 50 μl/well of 2 M H_2_SO_4_, and the absorbance was read at 450 nm on a SPECTROstart nano (BGM; LabTechnologies). The plotted values resulted from subtracting the absorbance from the control wells.

### Determination of IgT Total and Specific Antibody Levels

Direct and indirect ELISAs were performed to analyze the total and hemocyanin-specific gut mucus IgT levels, respectively, as previously described (46). The specificity of the customized polyclonal anti-GSBIgT antibody used in this study has been previously validated by Western blot and ELISA (46). Briefly, to assess the total IgT here, 96-well plates were coated with fish gut mucus diluted 1:6 in carbonate/bicarbonate solution and incubated overnight at 4°C. Similarly, to measure the hemocyanin-specific IgT level, 1:5 dilutions of fish gut mucus from the naive, hemocyanin-immunized, or pre-immune animals as control were added to a hemocyanin precoated flat-bottomed ELISA plates. Then, each assay was followed by the addition of a customized polyclonal anti-seabream IgT (GeneScript) and an anti-rabbit IgG peroxidase antibody produced in goat (Sigma-Aldrich, Spain), both diluted at 1:1,000. Finally, the chromogen TMB (Sigma-Aldrich) was added, and the plates were incubated at 22°C. The reaction was stopped with the addition of 50 μl/well of 2 M H_2_SO_4_, and the absorbance was read at 450 nm on a SPECTROstart nano (BGM; LabTechnologies). The plotted values resulted from subtracting the absorbance from the control wells.

### RNA Extraction and Gene Expression Analysis

Total RNA was extracted from the hindgut of control or treated (vaccinated or not) fish (n = 6 fish/treatment) with TRIzol reagent (Invitrogen) following the manufacturer’s instructions and quantified with a spectrophotometer (NanoDrop, ND-1000). The RNA was treated with DNase I, amplification grade (1 U/mg RNA; Invitrogen), to remove genomic DNA traces that might interfere with the PCRs. Subsequently, the SuperScript IV RNase H reverse transcriptase (Invitrogen, USA) was used to synthesize first-strand cDNA with oligo-dT18 primer from 1 µg total RNA, incubated at 50°C for 10 min. The b-actin (*actb*) gene was analyzed for sample content standardization using a semiquantitative PCR with an Eppendorf Mastercycle Gradient Instrument (Eppendorf). Briefly, the reaction mixtures were incubated for 2 min at 95°C, followed by 35 cycles of 45 s at 95°C, 45 s at the specific annealing temperature, 1 min at 72°C, and finally 10 min at 72°C. In the same samples, the expression levels of the genes coding for the pro-inflammatory cytokines *il1b* and *cox2* or the mucosal markers *imuc*, *muc13*, and *ight* were analyzed by real-time PCR performed with a QuantStudioTM 5 Flex instrument (Applied Biosystems) using SYBR Green PCR core reagents (Applied Biosystems). The reaction mixtures were incubated for 10 min at 95°C, followed by 40 cycles of 15 s at 95°C, 1 min at 60°C, and finally, 15 s at 95°C, 1 min at 60°C, and 15 s at 95°C. After verifying each primer pair amplification efficiency and single peak melting curve presence, the appropriate references were selected based on the average M value. Thereafter, the relative expression of each target gene was corrected by the content of two reference genes, the 40S ribosomal protein subunit 18 (*rps18*) and the subunit 11 (*rps11*) in each sample using the comparative cycle threshold method (2^-ΔΔCt^) (47). The gilthead seabream-specific primers used as targets and housekeeping genes are listed in **Table 1**. In all cases, each PCR was performed in duplicate with three technical replicates each.

**Table 1:** List and GeneBank accession number of primers used for qPCR analysis of seabream samples.

Gene	Name	Sequence (5’-3’)	Accesion number
*il1b*	F2	GGGTCTGAACAACAGCACTCTC	**AJ277166**
	R3	TTAACACTCTCCACCCTCCA	
*cox-2*	F2	CATCTTTGGGGAAACAATGG	**AM296029**
	R2	AGGCAGTGTTGATGATGTCG	
*imuc*	F	GTGTGACCTCTTCCGTTA	**JQ27712**
	R	GCAATGACAGCAATGACA	
*muc13*	F	TTCAAACCCGTGTGGTCCAG	**JQ27713**
	R	GCACAAGCAGACATAGTTCGGATAT	
*ight*	F	TGGCAAATTGATGGACAAAA	**FM145138**
	R	CCATCTCCCTTGTGGACAGT	
*rps18*	F	AGGGTGTTGGCAGACGTTAC	**AM490061**
	R	CTTCTGCCTGTTGAGGAACC	
*rps11*	F1	GGCGTCAACGTGTCAGAGTA	**NM_213377**
	R1	GCCTCTTCTCAAAACGGTTG	

### Intestine Histological Analysis

Hindgut samples (n = 6) from each experimental group were fixed in 10% buffered formalin solution and processed using routine methods. Subsequently, they were embedded in paraffin at 60°C to obtain a microtome cross-section with a thickness of 5 μm that was stained with periodic acid shift-alcian blue (PAS-A). The slides were mounted using approved substitute chemicals and subjected to optical microscopic evaluation. The abundance of goblet cells and intraepithelial lymphocytes was assessed.

### DNA Extraction, PCR Amplification, Amplicon Library Construct and Sequencing

Genomic DNA was extracted from pooled intestine samples (n = 6 fish/treatment) using the TriSure method (Bioline). The quality and integrity of the extracted DNA were checked on 1% (w/v) agarose gel stained with GelRed Nucleic Acid Stain 20,000x (InTRON Biotechnology, Seoul). The DNA concentration and purity were quantified fluorometrically using the Qubit™ dsDNA HS assay kit (Thermo Scientific). The DNA samples were stored at -20°C, and 30 ng were used for subsequent analyses. Libraries were constructed with 2 × 300 bp paired-end sequencing in the Ultrasequencing Service of the Bioinnovation Center of the University of Málaga, Spain, on an Illumina MiSeq platform (Illumina). Briefly, Illumina paired-end sequencing was carried out using the sense forward (341F) 5′-CCTACGGGNGGCWGCAG-3′ and (805R) 5′-GACTACHVG GGTATCTAATCC-3′ reverse primers targeting the variable regions V3–V4 regions of the 16S rRNA gene. After removing the Illumina barcodes and demultiplexing, the readings were combined using the Mothur software package (1.39.5 version). In addition, the assembled readings were filtered, excluding readings lower or higher than 80–2,000 bp long. Besides, non-specific PCR amplicons and singleton sequences were excluded in the subsequent analyses. All processing was done using the Mothur pipeline. The Mothur script was used to detect and remove chimera against the reference database, and the remaining representative non-chimeric sequences were then subjected to taxonomic assignment against the Greengenes 16S database (48), with 97% 16S similarity as the cutoff and clustered into operational taxonomic units (OTUs). The sequencing depth level was determined by the rarefaction curves obtained by plotting the number of observed OTUs against the number of sequences and Good’s coverage coefficient. The alpha diversity was estimated based on Shannon–Wiener, Chao1, and Simpson indices to determine taxonomic and phylogenetic structure diversity.

### Functional Metagenome Prediction and Analysis

PICRUSt (version 1.1.3) was used to predict the putative metagenome functional profiles of intestines from vaccinated vs. naive fish using the previously obtained 16S rRNA sequencing reads (49). The resulting metagenome inferences were entered into the Greengenes database (version 13.5), and the metagenome prediction of bacterial communities was conducted using the calculated data set after normalizing the 16S rRNA copy number. Nearest Sequenced Taxon Index (NSTI) scores for evaluating the accuracy of predicted metagenomes were categorized with the Kyoto Encyclopedia of Genes and Genomes (KEGG) pathways database (50) and bacterial functional profiles until KEGG modules level 3 were compared.

### Statistical Analysis

The microbiome data were analyzed by Good’s coverage, rarefaction curves, and alpha diversity indices, including Chao1 richness estimation and community diversity (Shannon index and Simpson) with Microbiome eAnalyst web platform (51). Normality (Shapiro–Wilk) and homogeneity of variance (Levene’s test) were performed, and the statistical significance of growth data and alpha diversity paired comparisons were determined by Student’s t-test (p < 0.05). All tests were performed with XLSTAT software. DESeq2 software was used to test for differential representation of OTUs by treatment (p < 0.01). The STAMP (Statistical Analysis of Metagenomics Profiles) was used to analyze the differential abundance of modules based on the gut sections and diet using ANOVA multiple-comparison test with *post hoc* Tukey–Kramer test (p < 0.05). The results from the gene expression, Ig levels, goblet cell count, and antimicrobial humoral activities were analyzed by two-way ANOVA and *post hoc* Tukey’s to determine the differences among groups. Note that in some figures, the number of individuals was lower than the established (n = 6) due to technical issues. Then, we ran again each data set using weighted and unweighted means, and the robustness of our data was not affected. The critical value for statistical significance was established at p ≤ 0.05. All statistical analyses were carried out using the GraphPad Prism 8.04 software.

## Results

### Oral EE_2_ Exposure Strongly Disrupts the Seabream Hepatic Function Without Mirroring It in the Gut Cellular Immune Function

**Figure 2:**
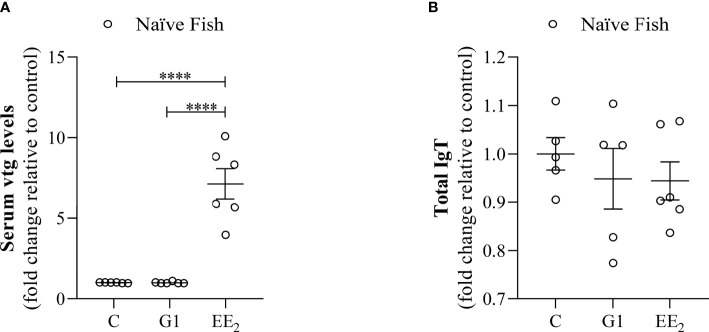
Dietary addition of xenoestrogen impairs vitellogenin but not total IgT production. Serum or intestinal mucus from gilthead seabream which had been treated with G1 or EE2 (5 μg/g food) for 43 days were assayed for: **(A)** serum vitellogenin (Vtg) level. **(B)** Intestinal mucus total immunoglobulin T (IgT). Both parameters were assessed with a double specific antibody procedure by ELISA. Representative ± SEM are shown (n = 6). The asterisks denote statistically significant differences after Student’s t-test between each treated and the untreated groups. ****(p<0.001).

Following our established estrogen-feeding model, the diet was supplemented with the G1 agonist or a strong xenoestrogen (EE_2_) and fed continuously for 84 days as described in detail (**Figure 1**). The continuous action of both treatments did not produce any fish mortality or abnormal behavior during the whole experimental period. However, after 42 days of constant EE_2_ oral intake, a significant (p < 0.001) estrogenic-mediated endocrine disruption was found after evaluating the VTG level from the blood serum (**Figure 2A**). On the contrary, the continuous administration of the G1 receptor agonist within the same period did not affect the VTG production supporting the notion that the action of EE_2_ triggers specific disruption. At the same time, the hindgut’s local adaptive immune response was not affected by any dietary supplementation, as demonstrated by the invariant yet modest levels of total mucus IgT recorded in both treated groups and the control fish (**Figure 2B**).

### Immunological Priming Reduces the Excessive Enzymatic Activity Triggered by EE_2_ in the Fish Systemic Response

**Figure 3:**
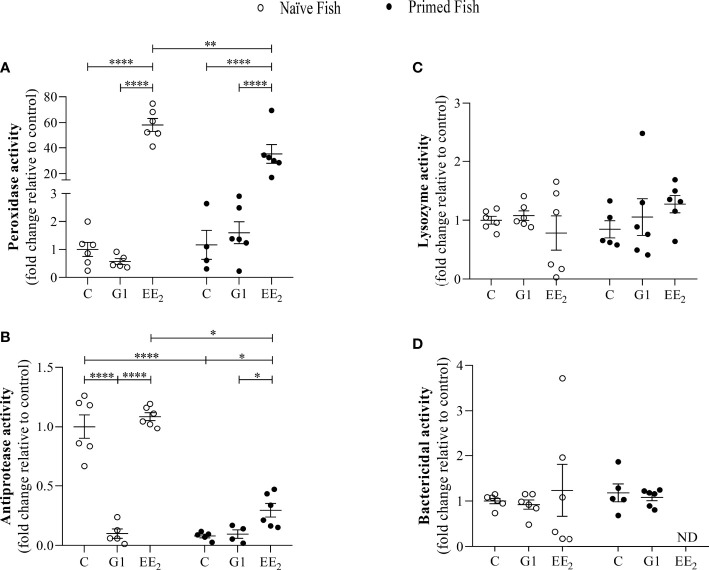
17α-ethinylestradiol (EE2) exposure and further immune priming modulates the antimicrobial humoral activities in the serum of gilthead seabream. The fish had been orally treated with G1 or EE2 (5 μg/g food) for 43 days and either immune primed with KLH or not (naive) on the sampling day. **(A)** Peroxidase, **(B)** Lysozyme, **(C)** Antiprotease **(D)** Bactericidal activity. Data represents means ± standard error (n = 6). Asterisks denote statistically significant differences between the groups. *(p<0.05) **(p<0.01) ****(p<0.001).

The magnitude of some major biochemical indicators known for impacting the cellular and defense functions in seabreams was assessed. After 1 day post-immunization, at 43 days of continuously treating the fish with the G1- or EE_2_-supplemented feed at naive or immunized conditions, a selected panel of proteolytic and bacteriolytic assays such as peroxidase, antiprotease, lysozyme, and bactericidal activities in the serum was collected as indicators. Interestingly, the naive fish treated with EE_2_ showed a markedly significant (p < 0.001) increase in the peroxidase activity relative to the control fish (**Figure 3A**). Unexpectedly, we observed that the priming immunization of the vaccine resulted in a significant (p < 0.01) decrease of the peroxidase activity in the EE_2_ group, even though the response remains significantly (p < 0.05) higher than that in the immunized controls. For the antiprotease response, a similar significant (p < 0.05) effect was also recorded at the EE_2_-treated group between the naive and immunized conditions, while the G1 treatment of the naive condition produced an opposite response by producing a significant (p < 0.001) inhibition (**Figure 3B**). At the same time, the lysozyme and bactericidal activities did not show any significant variations at any stage (**Figures 3C, D**).

### G1 and EE_2_ Exposures Hardly Alter the Antimicrobial Activities in the Gut Mucus Interphase

**Figure 4:**
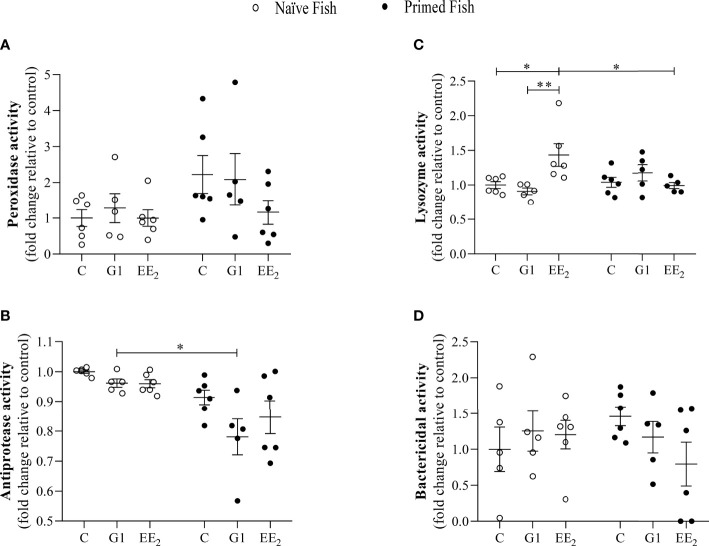
The gut mucus antimicrobial humoral activity is seldom affected by the presence of 17α-ethinylestradiol (EE2) or KLH-immune priming in gilthead seabream. The fish had been orally treated with G1 or EE2 (5 μg/g food) for 43 days and either immune primed with KLH or not (naive) on the previous day. **(A)** Peroxidase, **(B)** Lysozyme, **(C)** Antiprotease **(D)** Bactericidal activity. Data represent means ± standard error (n=6). Asterisks denote statistically significant differences between the groups. *(p<0.05) **(p<0.01).

Furthermore, to explore the constitutive gut mucus interphase as influenced by continuously feeding the seabreams with G1 or EE_2_, the biochemical assays previously described in the serum were conducted. We observed that the intestinal mucus of the animals exposed to either G1 or EE_2_ hardly showed any significant changes in either the peroxidase activity or the bactericidal capacity among the exposed groups and between the two conditions *per se* (**Figures 4A, D**). Although the gut mucus lysozyme present in the naive fish dietary administered with EE_2_ showed a dual effect by significantly (p < 0.05) increasing its activity level within the naive initial stage but resulting in a significant (p < 0.05) decrease after priming (**Figure 4C**). The antiprotease activity showed a significant variation resulting from treating the fish orally with the G1 agonist and comparing the response between the naive and the immunized conditions (**Figure 4B**).

### The Intestinal Level of Inflammatory Mediators Was Affected by the Oral Administration of EE_2_ and Can Be Reversed Through Vaccination

**Figure 5:**
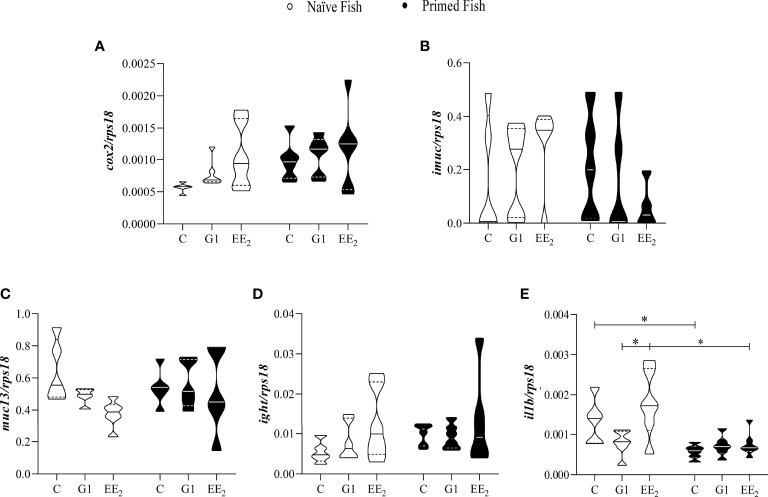
The relative immune gene expression in the gut of gilthead seabream in response to the exposure in either G1 or EE2 under naive or immune primed condition minimally change. The fish had been orally treated with G1 or EE2 (5 μg/g food) for 43 days and either primed with KLH or not (naive) on the previous day. Gene expression levels **(A)** cyclooxygenase-2; **(B)** intestinal mucin; **(C)** mucin-13; **(D)** immunoglobulin T heavy chain; **(E)** interleukin-1beta are related to the expression of the 40S ribosomal protein subunit 18 (rps18). The violin graphs visualize quantitative and qualitative attributes of the samples comprised in each group. The mean is represented by the central solid line on each plot (n=6). Asterisks denote statistically significant differences between the groups. *(p<0.05).

An accurate understanding of how the oral administration of G1 and EE_2_ reconfigures the immune system in the seabream is crucial to comprehend its disruptive effect. To address this, on day 43 of the trial, we profiled and quantified five critical inflammatory mediators, *cox2*, *imuc*, *muc13*, *ight*, and *il1b* at the transcriptional level. Differences in the expression pattern of the different mediators were assessed by quantitative-PCR (qPCR) following a naive scheme and after receiving the vaccine priming immunization. The mRNA transcriptional levels of the inducible inflammatory mediators *cox2* and *imuc* and the constitutive *muc13* and *ight* genes revealed subtle, yet not significant, changes in their expression at the naive condition (**Figures 5A–D**). At the same time, naive animals fed with EE_2_ showed a significant (p < 0.05) increase in the transcription level of *il1b* when compared to the G1-treated group. Furthermore, as seen in the previously analyzed parameters, the effect of priming immunization on the naive EE_2_ enhanced the expression of *il1b* significantly (p < 0.05) but eventually reverted to similar basal levels as those recorded in the untreated group (**Figure 5E**).

### Extended Oral Administration of EE_2_ Alters the Actively Secreting Mucus Cells

**Figure 6:**
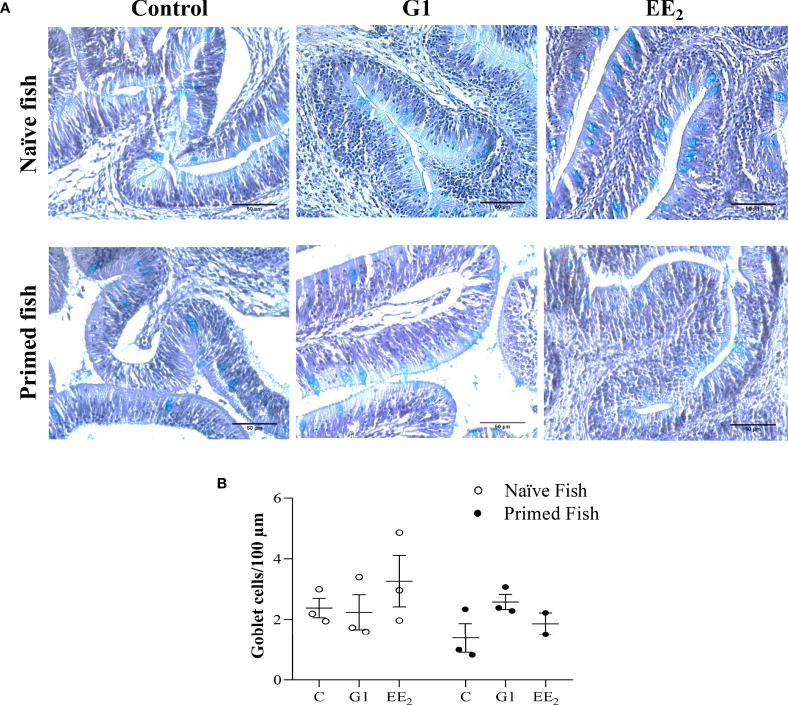
Oral exposure of G1, EE2 and immune priming induce structural changes along the hindgut of gilthead seabream. The fish had been orally treated with G1 or EE2 (5 μg/g food) for 43 days and either immune primed with KLH or not (naive) on the previous day. **(A)** Representative histomorphology image of PAS-A-stained hindgut longitudinal sections with neutral and acid staining goblet cells highlighted in light blue. Besides, note the high number of IEC infiltrates present in the lamina propria on both G1 and EE2 conditions. (n = 6; Scale bar = 50μm). **(B)** Representative average histological quantification of the mean number of PAS-positive goblet cells on three randomly selected slides per treatment and condition in duplication.

To further validate the positive effect of the immunization as previously observed on seabream expressing toxic effects after receiving a continuous oral dose of EE_2_, we analyzed the gut of the treated animals histologically under naive and immunized conditions. After 43 days of trial under naive conditions, we noted an increase in the volume and number of interepithelial lymphocytes (IELs) at the lamina propria and the PAS-A-stained (light blue) cells throughout the mucosal fold height architecture of EE_2_-treated fish. Likewise, this is morphologically consistent with neutral and acidic staining goblet cells, a specialized epithelial cell type that produces the main component of the mucus barrier. Unexpectedly, 24 h after the fish received the priming immunization of the vaccine, the previously altered goblet cells reverted to the basal state (**Figure 6A**). However, after quantifying the number of stained goblet cells on each treatment at either the naive or immunized stage using light microscopy, it was shown that the shift was not statistically supported. Yet, a decreasing trend can be observed (**Figure 6B**).

### Treating the Naive Seabream Orally With G1 and EE_2_, Together With the Vaccine Priming Immunization, Modulates the Gut Microbiota Communities

**Figure 7:**
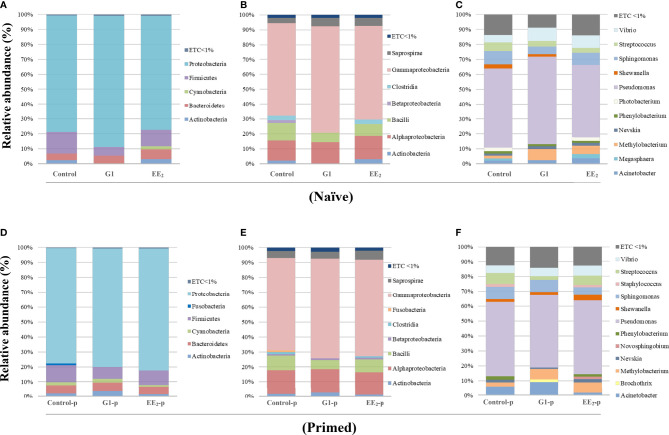
Relative abundance of the intestinal bacteria communities of gilthead seabream, at different taxonomic levels is affected by the oral G1 and EE2 exposure and further potentiated by immune priming. Phyla **(A, D)**. Class **(B, E)**. Genera **(C, F)**. *Left to right*, representing the received dietary treatment received (Control, G1, or EE2). *Up to down*; immune status (Naive or immune primed with KLH). ETC: average relative abundance < 1%, (n = 6).

The analysis of the different taxonomic levels in the three groups of naive fish revealed three dominant bacterial groups. The Proteobacteria showed the highest relative abundance (>75%) across all samples. It was followed by the phyla Firmicutes (6%–16%) and Bacteroidetes (4%–7%). Interestingly, the naive fish exposed to the action of the agonist G1 showed a significant abundance reduction of Actinobacteria and Firmicutes compared to the untreated or EE_2_-exposed animals (**Figure 7A**). At the class level, in naive fish, the dominant groups were the α- and γ-Proteobacteria in the naive fish, with the last class particularly more pronounced in the G1-treated fish (**Figure 7B**). Regarding the individual OTUs at the genus level, the three naive groups shared a high proportion of *Pseudomonas* with relative abundance ranging from 48% to 59%. Among the remaining genera, the most predominant genera in the three groups were *Vibrio*, *Streptococcus*, *Sphingomonas*, *Phenylobacterium*, *Nevskia*, *Methylobacterium*, and *Acinetobacter*, showing abundances between 1% and 9% but without any significant differences (**Figure 7C**). Unexpectedly, while the presence of *Shewanella* was recorded in the control and G1 naive groups, the EE_2_ treatment fully abrogated its presence causing a significant (p < 0.01) difference between groups. On the other hand, the results in immunized fish at the phyla, class, and genus levels (**Figures 7D–F**) mostly mirrored those previously reported for naive fish. However, significant differences in some OTUs were detected. At the phylum level, *Fusobacteria* was present only in the control group (**Figure 7D**), while the OTUs in the class β-Proteobacteria remarkably shifted in the gut microbiota abundance between the G1- and EE_2_-treated and immunized conditions (**Figure 7E**). The taxonomic analysis at the genus level revealed the largest variations in OTUs when comparing the core microbiota of untreated fish with the G1- and EE_2_-treated and priming immunized fish. *Brochothrix* in the G1-treated and *Staphylococcus* and *Novosphingobium* in the EE_2_-treated specimens were the most highly abundant genera between the naive and the vaccinated conditions (**Figure 7F**). Note that those sequences not assigned to any known microbial taxonomical classification were designated as ETC, which represented less than 1% of the entire individual data sets presented.

### Activation of the GPER1 Receptor Significantly Modulates the Gut Microbiome Structure

An average of 76,282.75 ± 12,605.17 reads was obtained from the samples analyzed. Furthermore, these sequences were successfully clustered in OTUs with 97% identity. The Good’s coverage estimations ranked between 99.98% and 99.99%, indicating an adequate sequencing depth. A random subsampling was conducted to normalize the data size to 42,494 reads that were assigned to 87 genera distributed among 14 phyla. The alpha diversity of the gut microbial community concerning its richness, diversity, and average evenness indices in every single sample within a treatment group, obtained from the next-generation sequencing (NGS) data, is summarized in **Table 2**. Oral treatment with G1 under naive conditions significantly (p < 0.05) decreased the Chao1 species richness and the Shannon diversity estimator indices for the gut mucosa-associated microbiota compartments. Interestingly, the Simpson index of diversity was decreased in the same G1-treated group, but this variation was not statistically supported. However, following the priming immunization variations on any of the three indices among groups, or changes between the control and the two experimental oral treatments were not observed.

**Table 2:** Alpha-diversity indices expressed as the richness, diversity, and average evenness (Chao1, Shannon and Simpson) of bacterial communities present in the hindgut of gilthead seabream (n=6 fish per condition) treated with G1 or EE_2_ (5 μg/g food) for 43 days either immune primed or not with KLH on the previous day’s (24 h) sampling.

Treatments	Immune Status	Chao1	Shannon	Simpson
Control (C)	Naïve	64.50 ± 7.59	2.53 ± 0.29	0.80 ± 0.06
G1		50.87 ± 8.51*	2.14 ± 0.20*	0.74 ± 0.07
EE_2_		60.25 ± 8.17	2.57 ± 0.17	0.83 ± 0.04
Control-Vac	Vaccinated	61.69 ± 5.98	2.56 ± 0.13	0.81 ± 0.02
G1-Vac		61.62 ± 9.96	2.65 ± 0.12	0.83 ± 0.02
EE_2_-Vac		66.12 ± 5.36	2.56 ± 0.22	0.81 ± 0.04

(*) denotes significant differences (p<0.05). For details refer to the statistics section.

### The Activation of the GPER1-Mediated Estrogen Action Modulates the Gut-Associated Microbiota Functional Activity

**Figure 8:**
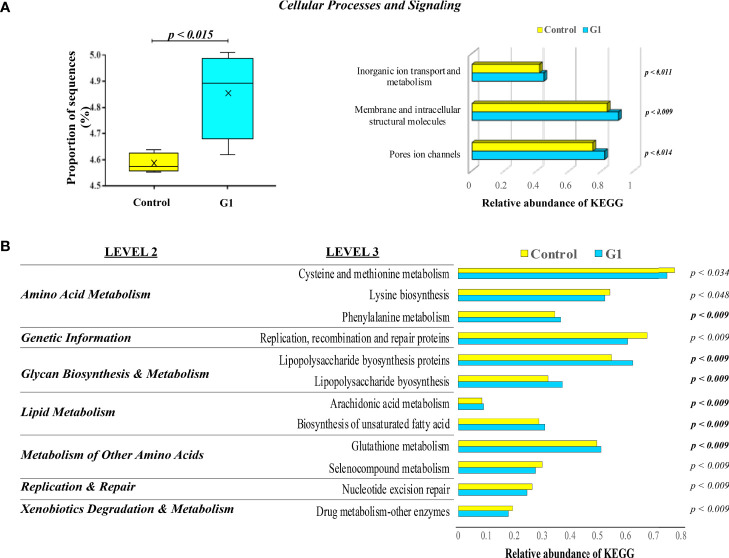
KEGG pathways and functional annotation analysis of gut mucosa-associated microbiota compartments in unvaccinated seabream. **(A)** Box plots representing the significant proportion of the relevant abundance of PICRUSt generated functions profile in the hindgut microbiota of gilthead seabream. The box represents the values between the 25^th^ and 75^th^ percentiles. The maximum and minimum values are represented by up and low caps. Treatment with the G1 containing diet led to a significant increase in the relative abundance of gene sequences influencing critical cellular processes and signaling terms. **(B)** Significant Kyoto Encyclopedia of Genes and Genomes (KEEG) pathways level 2 and 3 enriched by predicted genomes of microbiota from control fish vs. G1-orally treated animals for 43 days. The significant overexpression of each KEGG term in G1 compared to control fish fractions are indicated by the resulting p-value in bold analyzed by ANOVA with a *post-hoc* Tukey-Kramer multiple comparison test (p<0.05).

The changes in the presumptive functions of the gut microbiota of fish showing significant differences in their microbiotas in comparison with non-immunized untreated fish were examined by predicting the metagenomes using PICRUSt. The accuracy of the prediction was evaluated by computing the NSTI, and the mean of the samples was 0.048 ± 0.005, indicating a relatively good match to reference genomes [ideal NSTI ≤0.03 (52)]. The KEGG ortholog functional predictions showed a significant difference (p < 0.015) only between the unvaccinated control fish and those treated with G1. The increased abundances of the specific functional category associated with cellular processes and signaling of inorganic ion transport and metabolism, pore ion channels, and membrane and intracellular structural molecules are predicted with significant changes on the G1-fed group, with the latter category presenting the highest significance (p < 0.009) value (**Figure 8A**). For the control and G1 groups, the level 2 KEGG annotations revealed seven functional associations related to metabolic, replication and repair, and genetic information categories. By applying the more detailed annotation type, the KEGG level 3, 12 significantly different functional pathways were revealed between the two analyzed groups. Specifically, categories associated with phenylalanine metabolism, lipopolysaccharide (LPS) biosynthesis proteins, arachidonic acid metabolism, biosynthesis of unsaturated fatty acids, and glutathione metabolism were significantly more abundant in fish exposed to G1, whereas categories such as cysteine and methionine metabolism showed lower abundance in this same group (**Figure 8B**).

### Vaccination Does Not Alter the Production of Systemic and Mucosal-Specific Antibodies in G1- and EE_2_-Treated Fish

**Figure 9:**
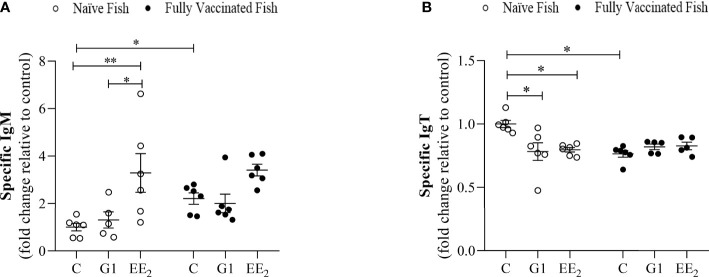
Vaccination differentially regulates the activity induced by the oral exposure to xenoestrogens on the systemic and mucosal antibody production in the gilthead seabream. The anti-KLH IgM and IgT levels (**A** and **B**, respectively) in fish dietary treated with G1 or EE2 (5 μg/g food) for 84 days and either vaccinated following priming and boosted doses of KLH or not (naive) are shown. Data were obtained in duplication by ELISA using independent biological samples and represent means ± standard error (n=6). Asterisks denote statistically significant differences between the groups. Data were analyzed by ANOVA with a *post hoc* Tukey-Kramer multiple comparison test, *(p<0.05) **(p<0.01).

The KLH-specific IgM and IgT levels in the serum and intestinal mucus were evaluated by ELISA on day 84 (end of the trial). The samples were collected from control, G1-treated, and EE_2_-treated seabreams, both naive and fully immunized after receiving the vaccine priming and booster doses on days 42 and 56, respectively. As expected, in naive animals, EE_2_ altered the production of natural antibodies that should have been overlapping with some epitopes of the specific IgM and IgT levels measured. However, the response followed a differentially expressed pattern between both targets. The IgM level in the naive EE_2_-fed animals produced a significantly (p < 0.001) elevated response compared to the control or G1-treated groups (**Figure 9A**). On the contrary, the specific IgT production in the gut mucosal secretion of naive fish at the same time point was significantly (p < 0.05) lower in both orally treated groups, suggesting an inhibitory effect on this parameter (**Figure 9B**). The specific systemic or mucosal anti-KLH was inversely differentiated from the naive condition. Although the vaccine did not trigger any significant enhancement in any group, it displayed a more stable effect in the control group when compared to the naive condition. Furthermore, no effect was observed at the end of the trial in the vaccinated and orally treated specimens with either the G1 agonist or the strong disruptor EE_2_.

## Discussion

In farmed teleost fish, the proper crosstalk between the commensal bacteria residing in the mucosa and the immune cells within the mucosal interphase is essential for controlling and preventing major immune disorders (53, 54). This link highlights the critical importance of cautiously monitoring the immune interventions in the presence of exogenous elements from either a spontaneous or planned artificial origin (e.g., environmentally generated EDCs or vaccination) that may alter such delicate crosstalks. Yet, in fish, the underpinning mechanisms and the key actors that govern the host–microbe intercommunication following the effect of combining the EDC and vaccination in a single setting are poorly understood.

Using our proven estrogen-feeding model and a two-step vaccination program, we found that the systematic oral intake of a moderate dose of G1 or EE_2_ promotes diverse physiological alterations that can potentially contribute to developing pathological profiles that are detrimental to exposed individuals. The fact that estrogens generate reactive oxygen species (ROS), induce peroxidation, and play a key modulatory role in mammals and fish immune systems is not new (34, 55). In our model, the observed endocrine disruption is attributed to the upregulation of hepatic signaling saturation by EE_2_ that resulted in excessive VTG production. However, parallel mechanisms equally linked with EE_2_ involving divergent pathways associated with the estrogenic, antiandrogenic, thyroid, peroxisome proliferator-activated receptor γ, retinoid, and actions mediated through other nuclear receptors; steroidogenic enzymes; neurotransmitter receptors and systems could not be ruled out from participating in the response. Consequently, altering the systemic and local immune transduction pathways *via* non-genomic mechanisms is mediated by GPER1, as previously suggested (56). Indeed, evidence for host–microbe interactions in mammals supports GPER1 as one of the host factors responsible for crosstalk with microbiota (57). However, the role of the GPER1 agonist, the G1, in modulating the alpha diversity indices and generating a microbial dysbiosis highlighted by the shift of *Shewanella* and *Photobacterium* proportions in the gut of naive fish is reported here for the first time. Thus, we anticipate that after longer exposures to EDCs, dysbiotic events must be monitored to prevent possible metabolic dysregulation and changes to gut barrier function that can lead to diseases in exposed fish.

Cumulative evidence emphasizes the influence of the gut microbiota on humoral and cellular vaccine responses (58, 59). Therefore, several studies have attempted to identify the core microbiota of gilthead seabream (60–62). Despite being not completely established, most studies have agreed with our findings on putting forward Proteobacteria, Firmicutes, Bacteroidetes, and Actinobacteria as the dominant microbial phyla present in the gut. However, these results are not surprising. From the trillions of commensal microorganisms that constitute the intestinal microbiota of most vertebrates, Firmicutes and Bacteroidetes predominate and represent ~90% of the total (63). However, the subtle but significant shifts observed in the relative abundance of Actinobacteria and Fusobacteria under the naive condition in response to the vaccination regardless of the treatment prompted our attention.

In our study, the endocrine disruption generated by EE_2_ altered (at least to some extent) most of the humoral and cellular parameters screened in the treated naive fish, although this did not trigger a distinct inflammatory pattern as determined by the lack of significant upregulation of inflammatory mediators at the intestinal mRNA or the cellular resolution. Nevertheless, the EE_2_ treatment generated an increase in size and number of goblet cells, promoted the IEL infiltration to the lamina propria, and perhaps also contributed to a general redistribution of leukocytes among the entire system. Furthermore, it is accepted that EE_2_ can interact with other components of the endocrine pathway, e.g., ER corepressors or coactivators, and modulate the activity of the enzymes involved in steroid conversion, e.g., aromatase CYP19 converts testosterone to E_2_ (64). This conversion leads to stimulation or inhibition of endogenous hormone biosynthesis. Besides, EDCs like the EE_2_ can bind to circulating hormone-binding proteins and disrupt the local endogenous hormonal balance expressing cortisol that ultimately regulates neuroimmunoendocrine circuitries, elicits stress-induced immunosuppression, and contributes to allostatic imbalances (65). In any case, in our study, vaccination significantly contributes to the immune restoration and cellular and biochemical homeostasis in fish 24 h post priming immunization on day 42 of the trial. Presumably, the positive effect of the vaccine has over the estrogenic disruption by EE_2_ was mediated through the rapid release of specific commensal microbial factors after a single priming immunization dose. Previously, we have demonstrated that damage-associated molecular patterns released from dying cells mediate alum adjuvant activity when combined with KLH (39). However, in this study, the role of alum as adjuvant remains elusive, and future studies are required to understand its associated mechanisms. In mammals, it has been demonstrated that estrogen generates changes in the gut microbiota and might promote the enrichment of bacteria associated with immune regulation (66). In addition, the diversity of the gut microbiota has been shown to influence systemic estrogen levels through enzymatic and immune pathways (67). Although, in the present study, an increased modulation of the adaptive immunity expressed as specific IgM and IgT levels by the end of the trial in any group was unsupported.

The alpha diversity indices (richness, diversity, and average evenness) of the gut microbial community in every pooled sample within a treatment group only showed significant differences in fish from the G1 naive treatment. This indicates that the bacterial diversity in these communities was mostly covered regardless of the diet. In the gut microbiota of fish, including the gilthead seabream, Actinobacteria is a predominant low-represented phylum (60, 68). In the present study, Actinobacteria was completely abrogated at the phyla and class levels in the G1-treated naive fish when compared to the control or EE_2_ treatments. However, in the same group of the vaccinated fish, Actinobacteria was always present among the bacterial OTU repertoires. Previous studies have suggested that mice treated with G1 and subjected to a mucosal bacterial infection have reduced bacterial burden (69). Moreover, the low bacterial burden generated local levels of the neutrophil-recruiting chemokine *cxcl1* and the inflammatory cytokine *il1b*, like our results. Therefore, we hypothesize that, in our study, G1 also promoted a diversity reduction through the enhancement of the endothelial and epithelial gut–blood barrier integrity and increased permeability through the reorganization of dynamic structures like the tight junctions in a way that the paracellular permeability can adapt to the required characteristics of diffusion restriction mediated by the external stimuli. On this basis, we speculate that G1 has a strong potential utility as a viable solution to enhance specific bacteria releasing variated lytic compounds (70), as probiotic (71) or by producing secondary metabolites that have critical functions in the physiological, biochemical, or defensive role within their host (72).

With respect to the vaccine response, our results indicate that at the genus level, *Staphylococcus*, *Brochothrix*, and *Novosphingobium* may have driven many of the differences observed in the response to vaccines. In particular, *Novosphingobium* is well known for its capacity to degrade a wide range of environmental pollutants, with a particular emphasis on the removal of estrogen (73). These experimental findings support the hypothesis that differences in estrogen metabolism are associated with variability in the gut microbial diversity. *Novosphingobium* also assists in the metabolism of nitrogenous compounds and sulfanilic acid, so its ability to metabolize several xenobiotics has also been described (74, 75). Besides, the relationships between gut microbial richness, systemic and fecal estrogens, and beta-glucuronidase activity have been demonstrated (67). Based on these concepts, the term estrobolome was coined to define the gene repertoire of the gut microbiota capable of metabolizing estrogens (28, 76). However, just recently, the wider term endobolome was also suggested to address the group of gut microbiota, genes, and pathways involved in the metabolism of any EDC, including the target of this study, the EE_2_ (29). Together, these concepts explain why a balanced bacterial composition is a key player in eliciting downstream gene activation and triggering intracellular signaling cascades to maintain intestinal and distal mucosal sites immunity and homeostasis.

Meanwhile, the PICRUSt analysis of our study showed that seabream fed with the G1-containing diet acquired a significant increase in the relative abundance of gene sequences influencing critical cellular processes and signaling terms. Specifically, the categories associated were related to ion channels, membrane, intracellular molecules, inorganic ion transport, and metabolism. At the immune level, ion exchange between intracellular and extracellular spaces is the primary mechanism for controlling cell metabolism and signal transduction (77). This process is mediated by the predicted overexpressed ion channels and transporters on the plasma membrane or intracellular membranes that surround various organelles in response to environmental stimuli like the one generated by the presence of EE_2_. In fish, we have already demonstrated the importance of the ion channels in mucosal tissues on the transportation of calcium between the extracellular and intracellular environment to initiate inflammatory processes mediated through the transient receptor potential vanilloid 4 (TRPV4) (78). EE_2_ potentiates TRPV1 as an activated ionotropic membrane receptor coupling with mitochondrial function and cell survival in injured immunocytes following an oxidative manner (79). G1-treated fish also showed predicted functions associated with LPS biosynthesis and arachidonic acid (ARA) metabolism. ARA is an n-6 essential fatty acid present in the membrane phospholipid released by phospholipase A2 that further is used to produce eicosanoids. Recent studies have elucidated that ARA influences innate immunity *via* regulating the development and differentiation of innate immune cells and the function of the intestinal barrier (80). Therefore, in our study, ARA generation in the intestine of seabream may directly affect the gut immune environment by enhancing the crosstalk between the intestinal epithelial barrier and the professional immune cells inhabiting the same. In addition, it has been observed that the proliferation of Firmicutes inhibits the proliferation of LPS-producing bacteria, reducing the transfer of potential bacterial inflammatory products to the liver (81), thus exacerbating the hepatic toxicity generated by the presence of the EDC. Similarly, the microbiota of G1-treated fish in our study showed a reduction in Firmicutes. Perhaps, this effect was related to the increased abundance of LPS biosynthesis. Also, the changes observed in the gut microbiota of fish from the G1 treatment are associated with ARA metabolism. This opens the possibility that the microbiota in the gut exerts a potential exogenous effect in promoting ARA release since fish lack the ability to synthesize polyunsaturated fatty acid (PUFA) and require preformed dietary PUFA from linolenic and linoleic acids such as ARA for their normal growth and development (82).

In summary, we have provided evidence of the beneficial effects of the gut bacteria, which overall appear capable of processing endogenous estrogens or xenoestrogens such as EE_2_ through the endobolome. Moreover, these bacteria, together with a vaccination scheme, seem to provide robust supplementary support on the health management of the cultured seabream subjected to the action of EDCs, despite lacking a significant enhanced specific antibody production. However, this knowledge requires extensive further studies to validate the proposed concepts functionally.

## Data Availability Statement

The original contributions presented in the study are publicly available. These data can be found here: National Center for Biotechnology Information (NCBI) BioProject database under accession number PRJNA749661. The BioProject is permanently accessible through the following link: https://www.ncbi.nlm.nih.gov/bioproject/?term=PRJNA749661.

## Ethics Statement

This study was carried out in strict accordance with the recommendations under the Guide for Care and Use of Laboratory Animals of the European Union Council (2010/63/ UE). The number of animals used was determined following a highly restricted *f* size *a priori* effect established at the 0.05 a-error probability on the power analysis, determined as previously suggested (37). The Bioethical Committee approved the experimental protocol of the IEO (reference REGA ES300261040017) and the “Consejería de Agua, Agricultura y Medio Ambiente” of the “Región de Murcia”, Spain (approval number A13160508). Written informed consent was obtained from the owners for the participation of their animals in this study.

## Author Contributions

AG-A conceived and designed the experiments. PC, IC, VG, EC-P, and IC-O performed the experiments. EC-P, MM, EM-M, JG-V, and AG-A analyzed the data. PC original draft preparation, and JG-V reviewed and editing. All authors contributed to the article and approved the submitted version.

## Funding

This study was funded by the Ministerio de Ciencia e Innovación and FEDER (AGL2017-85978-C2-1-R). The Fundación Séneca (CARM) (19883/GERM/15). Nord University Access Fund covers the OA publication cost.

## Conflict of Interest

The authors declare that the research was conducted in the absence of any commercial or financial relationships that could be construed as a potential conflict of interest.

## Publisher’s Note

All claims expressed in this article are solely those of the authors and do not necessarily represent those of their affiliated organizations, or those of the publisher, the editors and the reviewers. Any product that may be evaluated in this article, or claim that may be made by its manufacturer, is not guaranteed or endorsed by the publisher.
